# The Impact of Obesity on Reflux Recurrence Following Laparoscopic Anti-reflux Surgery: An Evidence-Based Systematic Review and Meta-Analysis

**DOI:** 10.7759/cureus.56981

**Published:** 2024-03-26

**Authors:** Faisal Nadeem, Ananya Singh, Muhammad Karim, Amir Khan, Salman Mirza, Syed A Kabir

**Affiliations:** 1 General Surgery/Bariatric Surgery, Walsall Manor Hospital, Walsall, GBR; 2 Laparoscopic Surgery, Maroof International Hospital, Islamabad, PAK; 3 General Surgery, Walsall Manor Hospital, Walsall, GBR; 4 Bariatric Surgery, Walsall Manor Hospital, Walsall, GBR

**Keywords:** anti-reflux surgery, fundoplication, obesity, gastroesophageal reflux disease (gerd), high bmi, weight, reflux symptoms

## Abstract

Gastroesophageal reflux disease (GERD) is frequently seen in the Western population. Laparoscopic anti-reflux surgery (LARS) is effective in managing this condition. Obesity is strongly associated with GERD, and with the rising rate of obesity, there is, therefore, a concurrently increasing frequency of LARS performed. We aim to review the outcomes of LARS in patients with obesity, including the recurrence of GERD symptoms and peri-operative complications. A systematic review and meta-analysis were performed for articles from June 1992 to June 2022. The literature was reviewed for outcomes of LARS in patients with obesity (BMI≥30). Eligibility criteria included specific BMI, study design, type of surgery, and outcomes. The recurrence of symptoms and peri-operative complications were assessed. Thirty-one studies were thoroughly reviewed. Nine studies (five retrospective and four prospective) were selected for meta-analysis using the Preferred Reporting Items for Systematic Reviews and Meta-Analyses (PRISMA) flow, which included 1,499 patients with obesity and 5,521 without. Laparoscopic Nissen fundoplication was the most common procedure performed. The recurrence of symptoms was significantly lower in patients without obesity (p=0.0001). There was no statistically significant difference between patients with and without obesity in peri-operative complications, re-intervention, and early return to theatres. A higher recurrence rate of GERD symptoms post-LARS was reported in patients with obesity. Further research is required to decrease such risks and propose different methods, such as weight loss prior to surgery or Roux-en-Y (R&Y) gastric bypass. Risks and benefits should be considered by clinicians prior to offering LARS to patients with obesity.

## Introduction and background

Gastroesophageal reflux disease (GERD) presents a significant challenge for medical practitioners, with its prevalence steadily escalating globally. Although precise prevalence figures remain elusive, estimates suggest rates of 10 to 30% among Western populations [1]. In response, laparoscopic anti-reflux surgery (LARS) has emerged as a definitive solution, offering an alternative to lifelong medical acid suppression therapy. This is particularly relevant for individuals enduring persistent or recurrent acid reflux despite optimal medical interventions. Laparoscopic Nissen fundoplication (LNF) is the most commonly performed procedure worldwide [2,3]. Furthermore, specific indications for LARS include non-compliant patients on high-dose acid suppression therapy and those deemed too young for lifelong medical regimens [4].

The escalating prevalence of obesity, doubling over the past three decades, according to the World Health Organization (WHO), has intensified the intersection of obesity and GERD. Pathophysiological explanations, supported by epidemiological studies, underscore the association between obesity and GERD. Obesity's contribution to increased intra-abdominal pressure diminished lower oesophageal sphincter pressure, and compromised gastric motility amplifies the prevalence of symptomatic GERD, with high BMI cohorts displaying triple the risk or more [5,6]. Consequently, the surge in obese GERD patients has led to a surge in LARS procedures for this demographic.

Paradoxically, LARS within this obese patient group has generated controversy due to conflicting outcomes reported by surgeons. A consensus on complications such as reflux recurrence, wrap slippage, migration, and post-operative herniation into the chest remains elusive [7,8].

This study aims to address these uncertainties through a systematic review and meta-analysis, scrutinizing post-LARS outcomes in obese patients with reflux disease. The primary focus is on the recurrence incidence of reflux symptoms post-LARS. Secondary objectives encompass the prevalence of peri- and post-operative complications, encompassing re-interventions such as endoscopic dilation or revision surgery and immediate post-operative returns to the operating room. Ultimately, this study seeks to guide the optimal treatment approach for this patient subset.

## Review

Materials and methods

This systematic review was done under the Preferred Reporting Items for Systematic Review and Meta-Analyses (PRISMA) guidelines [9]. A detailed search strategy was devised to identify relevant articles for inclusion in the study.

A meticulous search of prominent databases was conducted, including PubMed, Embase, MEDLINE, Ovid, the Cochrane Library, and Google Scholar. The search encompassed the period from June 1992 to June 2022, focusing on literature reporting outcomes of LARS in patients stratified by their Body Mass Index (BMI).

The search was facilitated by utilizing a set of targeted keywords. These keywords included obesity, obese, weight, BMI, gastroesophageal reflux disease (GERD), anti-reflux surgery, Nissen fundoplication, fundoplication, and reflux. These terms were skilfully combined to generate a comprehensive pool of search results. Two independent researchers (SAK and MK) reviewed the results meticulously to ensure accuracy and rigor.

To further enrich the pool of potential articles, the reference lists of the fetched articles were systematically examined, employing a manual hand-search approach.

Eligibility Criteria

Employing the Patient, Intervention, Comparators, Outcome, and Study Design (PICOS) framework as the basis for our search criteria, we formulated a set of comprehensive inclusion and exclusion criteria. These criteria have been precisely defined and are presented in detail in Table 1 for reference.

**Table 1:** PICOS framework, inclusion and exclusion criteria PICOS: Patient, Intervention, Comparators, Outcome, and Study Design, GERD: Gastroesophageal reflux disease

PICOS	Inclusion criteria	Exclusion criteria
Patient	Categorized as obese by the WHO criteria (BMI > 30), suffering from GERD.	Under the age of 18.
Intervention	Laparoscopic anti-reflux surgery (laparoscopic Nissen fundoplication, laparoscopic Toupet, laparoscopic anterior, or any posterior wrap).	Re-do surgery, open surgery, and bariatric procedures.
Comparison	Categorized as non-obese by the WHO criteria (BMI <30), suffering from GERD.	Under the age of 18 years.
Outcome	Primary: Recurrence (symptomatic). Secondary: Incidence of peri and post-operative complications in the form of re-intervention such as endoscopic dilatation or re-do surgery and return to theatre early.	N/A
Study design	Randomized controlled trials, controlled trials (e.g., non-randomized, historical controls), observational studies, and conference proceedings with sufficient data available. No restriction on language or region.	Animal studies.

The study encompassed papers that presented data on both obese and non-obese patients, classified according to the WHO BMI classification system [10]. In a more specific context, patients included were those with obesity (BMI > 30 kg/m²) undergoing LARS as their primary surgical intervention. Additionally, non-obese patients (BMI < 30 kg/m²) suffering from acid reflux and undergoing LARS for relief from GERD were also considered for inclusion.

The focal point of this study revolved around the recurrence of reflux symptoms, serving as the primary outcome measure. This assessment was conducted either subjectively through clinical symptom reporting or objectively utilizing established methodologies like pH studies, barium studies, oesophageal manometry, and oesophagoscopy (OGD). Supplementary outcomes comprised the occurrence of peri- and post-operative complications, entailing re-interventions such as endoscopic dilatation or re-do surgeries, along with instances of immediate post-operative return to the operating theatre. Both prospective and retrospective studies were included in the analysis.

However, specific exclusions were made in the paper selection process. This encompassed literature lacking the primary outcome of interest, expert opinions, reviews, studies about patients undergoing LARS for a second or third time, data pertaining to patients under 18, and conference abstracts lacking complete research papers. Furthermore, papers presenting the recurrence of reflux using non-validated scoring systems or methods were also excluded from the analysis due to the infeasibility of conducting a meta-analysis with such data.

Statistical Analysis and Quality Assessment

Following the rigorous guidelines established by the Cochrane Collaboration, the statistical analysis was conducted using Review Manager (RevMan) 5.4.1 software, a trusted tool developed by The Nordic Cochrane Collaboration. Additionally, the visualization of data trends was facilitated by creating forest plots using the same software.

Given the anticipated diversity in the data set, dichotomous data were scrutinized using the odds ratio (OR). Recognizing the inherent heterogeneity of the data, the analysis employed a Mantel-Haenszel random-effects model across all meta-analyses. The presentation of the pooled ORs was accompanied by their corresponding 95% confidence intervals (CI). Significance was established at a threshold of p < 0.05, while the 95% CI not spanning 1 was indicative of statistical significance.

Furthermore, the Newcastle-Ottawa Scale was used to holistically evaluate the quality of non-randomized cohort studies [11]. This evaluative tool encompasses a triad of domains: the methodological assessment of case selection, the comparability of cohorts, and the meticulousness of outcome assessment and follow-up. The quality of each study was quantified through a star scoring system, wherein a minimum of seven stars out of a possible nine signified a study of high quality.

This meticulous approach underscores the commitment to rigorous statistical analysis and stringent quality assessment, culminating in robust findings and comprehensive insights.

Heterogeneity and Publication Bias

To comprehensively evaluate the variability and potential sources of heterogeneity among the included studies, the I2 statistic was employed and reported for each meta-analysis. Spanning from 0% to 100%, the I2 values provide a spectrum of heterogeneity, with values closer to 0% indicating minimal diversity among the studies. This statistical parameter quantifies the proportion of variation attributed to heterogeneity rather than random chance.

A P-value below 0.05 indicated statistical significance as relevant and applicable. By employing these analytical tools, the study ensured a rigorous examination of heterogeneity and its impact on the collective findings, augmenting the overall credibility and reliability of the results.

Results

**Figure 1:**
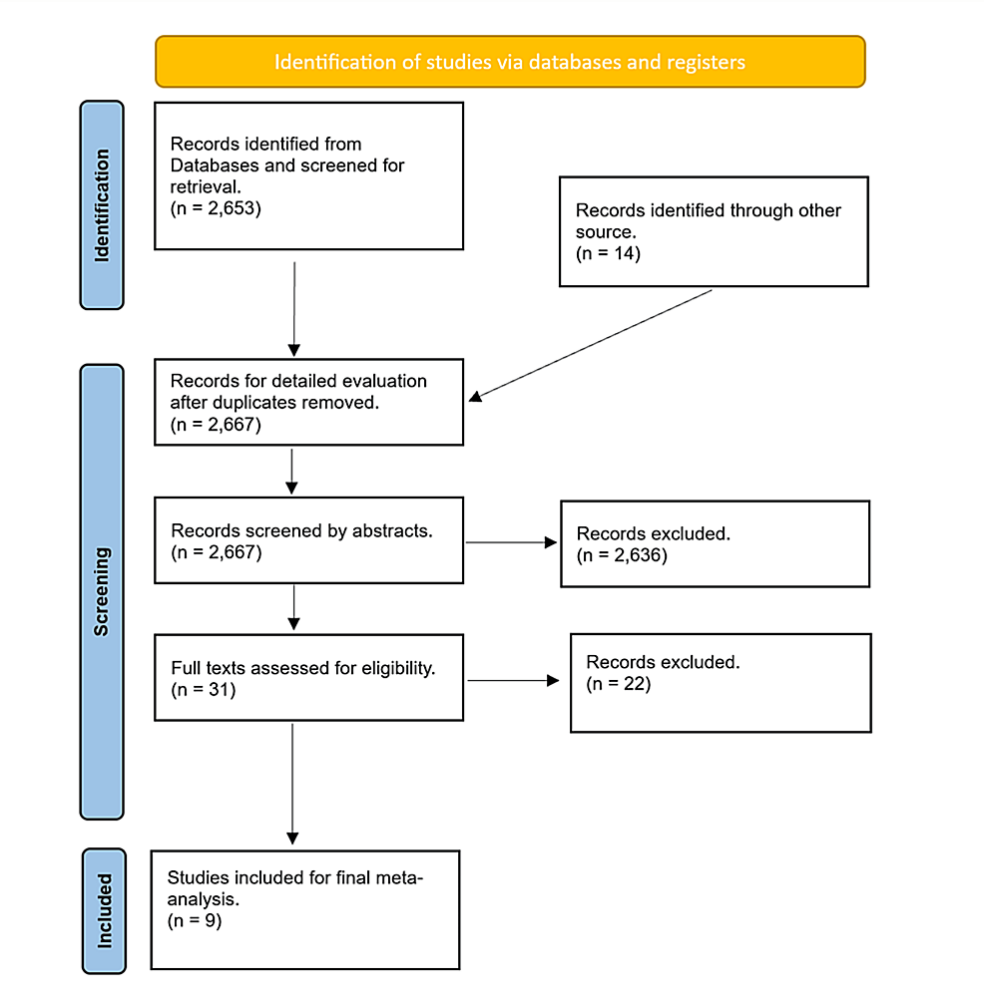
PRISMA flowchart for identifying eligible studies PRISMA: Preferred Reporting Items for Systematic Review and Meta-Analyses

Following an exhaustive process of identification and meticulous screening, a total of 35 studies were considered for inclusion in the initial evaluation. Ultimately, a subset of 13 cohort studies met the stringent criteria for inclusion in the comprehensive meta-analysis, as visually depicted in Figure 1. The subsequent statistical analysis was conducted on a substantial cohort of 7999 (8005) patients who had undergone LARS. Among this patient pool, 1753 individuals exhibited obesity, while the remaining 6246 participants were classified as non-obese. This robust dataset forms the foundation for the subsequent findings and insights.

Characteristics of Selected Studies

Among the final nine studies included in the analysis (as depicted in Figure 1), four were prospective cohort studies [12-15], while the remaining five were retrospective cohort studies [16-20]. The critical outcomes investigated in these studies are summarized in Table 2. Specifically, six studies assessed the recurrence of reflux symptoms following LARS [16,18-20], six studies reported perioperative complications [13-15,17-18], and an additional five studies focused on re-intervention cases involving re-do LARS [12-14,18-19]. Furthermore, four studies reported instances of re-intervention through endoscopic dilatation [12-14,19], three studies examined the conversion rate from initial laparoscopic to open procedures [12-13,19], and four studies investigated the occurrence of early return to the operating theatre post-operation [13,15,17-18]. The diverse outcomes examined across these studies collectively contribute to a comprehensive understanding of the subject matter.

**Table 2:** Studies included and their outcomes RR: Recurrence of reflux, PC: Perioperative complications, RE-HS: Re-intervention by re-do hiatal surgery, RE-ED: Re-intervention via endoscopic dilatation, CON: Conversion from laparoscopic to open, ERT: Early return to theatre

Study	Publication study year	Design of the study	Total number of cases (n)	Follow-up in months	BMI categories	Regrouping of BMI for meta-analyses	BMI (n)	Reported outcomes	Study quality score
Tekin et al. [12]	2011	Prospective cohort	1000	53	<25	<30	868	RR, PC, RE-HS, RE-ED, CON	7
					25–29.9	≥30-35	132		
					≥30	≥35	70		
Ng et al. [13]	2007	Prospective cohort	366	12	<30	<30	292	PC, RE-HS, RE-ED, CON, ERT	7
					≥30	≥30	74		
Winslow et al. [14]	2003	Prospective cohort	504	35	<25	<30	292	PC, RE-HS, RE-ED, ERT	7
					25–29.9				
					≥30	≥30	212		
D'Alessio et al. [15]	2005	Prospective cohort	257	26	<25,	<30	195	PC, ERT, OD	8
					25–29.9				
					≥30	≥30	62		
Tsuboi et al. [16]	2009	Retrospective cohort	145	77	<25	<30	135	RR	9
					25–29.9				
					≥30	≥30	10		
Telem et al. [17]	2014	Retrospective cohort	4231	1	<35	<35	3496	PC, ERT	7
					≥35	≥35	735		
Anvari & Bamehriz [18]	2005	Retrospective cohort	140	41.6	<30	<30	70	RR, PC, RE-HS, ERT	7
					≥35	≥30	70		
Schietroma et al. [19]	2017	Retrospective cohort	201	198	<25	<30	132	RR, RE-HS, RE-ED, CON	8
					25–29.9				
					≥30	≥30	69		
Andolfi et al. [20]	2017	Retrospective cohort	176	17	<30	<30	111	RR	7
					30–34.9	≥30	65		
					≥35				

Categories of BMI

Within our meta-analysis, we identified nine studies that explored our primary and secondary outcome variables in relation to patients' BMI categories. Out of these nine studies, five papers conducted comparisons based on BMI ranges: < 25, 25-29.9, and ≥ 30 kg/m² [12,14-16,19], while one study examined outcomes for BMI ranges of < 30, 30-34.9, and ≥ 35 kg/m² [20]. Additionally, three studies [13,17-18] employed a two-cohort design to compare outcomes for BMI < 30 kg/m² and BMI > 30 kg/m². A study by Telem et al. employed a two-cohort approach, categorizing patients into BMI groups of < 35 kg/m² and > 35 kg/m² [17]. Although this particular study did not directly compare outcomes between non-obese and obese patients as defined within the scope of our meta-analysis, it was deemed relevant to address the study objectives. Therefore, we conducted meta-analyses with and without this study to ensure unbiased results.

Type of Anti-reflux Surgery

The types of anti-reflux surgery (ARS) performed by the surgical units were documented in all selected papers included in our final analysis. Among these, four studies reported patients undergoing LNF [15,17-19], while three studies encompassed a mix of LNF and laparoscopic partial anterior fundoplication (LPAF) [12-15]. Only one study had a higher proportion of patients undergoing Laparoscopic Toupet/partial posterior fundoplication compared to LNF [16]. Additionally, one retrospective cohort study included a combination of LNF, LPAF, and Dor fundoplication [19]. Although we intended to perform a subgroup analysis to explore the impact of the ARS technique on reflux recurrence outcomes, limited reporting of procedure-specific numbers hindered our efforts. Overall, our final analysis indicated that LNF was the most commonly performed ARS technique.

Recurrence of Reflux (Primary Outcome)

Among the studies reporting a recurrence of reflux following LARS with corresponding BMI information [12-16,18-20], eight were initially considered. However, one study was excluded due to the use of non-dichotomous data or subjective

measures, such as direct questioning, to define recurrence [18]. Ultimately, four studies met our inclusion criteria for the final meta-analysis, reporting validated investigations confirming recurrence, supplemented by symptoms such as positive pH manometry and/or endoscopy findings or barium swallow [12,16,19-20]. The median follow-up duration across these studies was 35 months.

**Figure 2:**
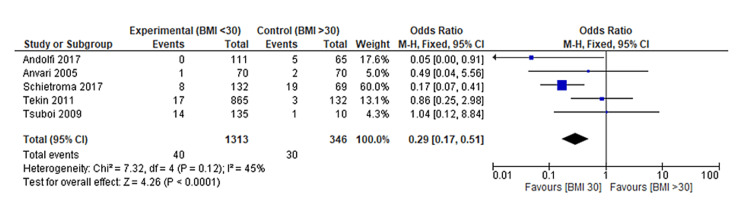
Forest plot on the outcome of recurrence of reflux symptom Tekin et al. [12], Tsuboi et al. [16], Anvari & Bamehriz [18], Schietroma et al. [19], Andolfi et al. [20]

**Figure 3:**
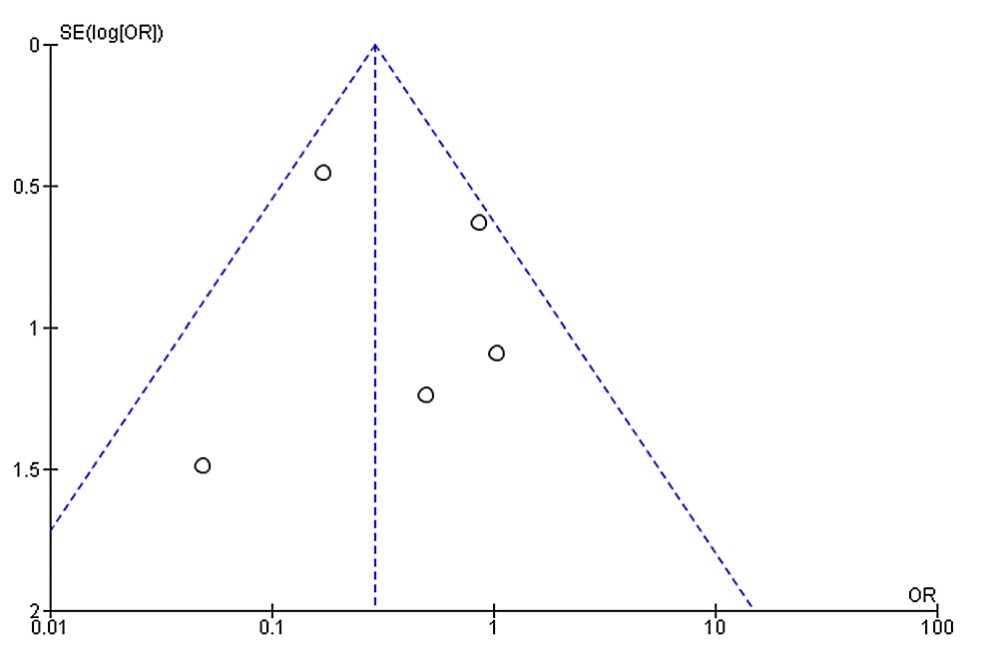
Funnel plots for the studied outcomes were symmetrical, suggesting the absence of publication bias on the outcome of the recurrence of reflux symptoms

The final meta-analysis revealed a significantly lower rate of recurrence of reflux symptoms in non-obese patients compared to obese patients following LARS (OR: 0.29, 95% CI: 0.17 to 0.51, p = 0.0001) (Figure 2). Furthermore, the funnel plot displaying a symmetrical distribution suggests the absence of publication bias (Figure 3).

Complications Associated With Laparoscopic Anti-Reflux Surgery

The occurrence of complications related to LARS was reported in six studies, encompassing both obese and non-obese patients [12-13,15,17-19]. These complications were categorized into intra-operative events, including bleeding, injuries to various organs (pleura, liver, spleen), and gastrointestinal perforations, as well as post-operative complications, such as wound infections, urinary tract infections, diarrhea, and chest-related issues (pneumonia, atelectasis, effusion, pulmonary embolism). Additionally, the analysis considered complications like deep vein thrombosis, cardiac events (atrial fibrillation, infarction, cardiac arrest), acute kidney injury, and wrap slippage necessitating early reoperation.

**Figure 4:**
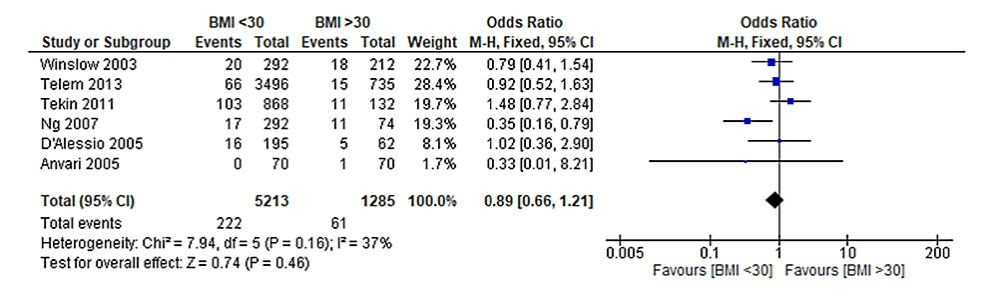
Forest plot on LARS-associated perioperative complications in non-obese and obese patients Tekin et al. [12], Ng et al. [13], Winslow et al. [14], D'Alessio et al. [15], Telem et al. [17], Anvari & Bamehriz [18] LARS: Laparoscopic anti-reflux surgery

**Figure 5:**
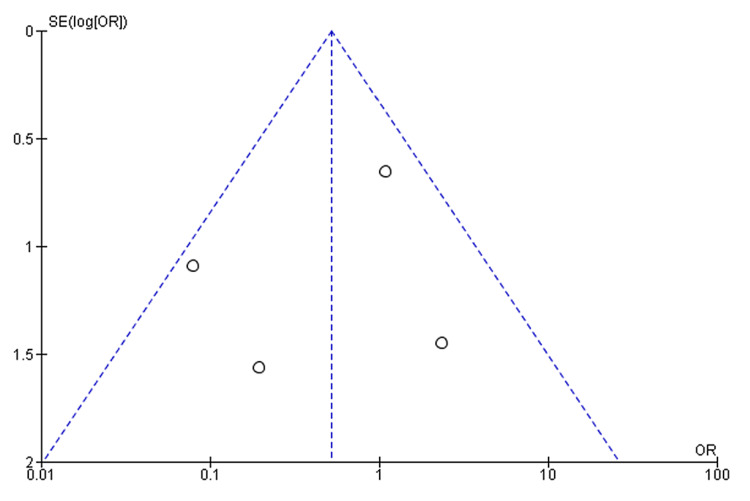
Funnel plots for the studied outcomes were symmetrical, suggesting the absence of publication bias on LARS-associated perioperative complications in non-obese and obese patients LARS: Laparoscopic anti-reflux surgery

Upon conducting our meta-analysis, we found no statistically significant difference in the occurrence of complications between non-obese and obese patients undergoing LARS (OR: 0.89, 95% CI: 0.66 to 1.21, p = 0.46) (Figure 4). This outcome aligns with the findings of a similar meta-analysis conducted by Telem et al. [17]. Furthermore, the funnel plot displaying a symmetrical distribution suggests the absence of publication bias (Figure 5).

Re-intervention and Re-do Surgery

**Figure 6:**
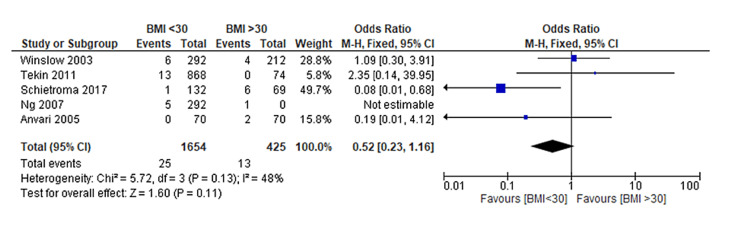
Forest plot on re-intervention by re-do hiatal surgery post-LARS in non-obese and obese patients Tekin et al. [12], Ng et al. [13], Winslow et al. [14], Anvari & Bamehriz [18], Schietroma et al. [19] LARS: Laparoscopic anti-reflux surgery

Upon conducting a meta-analysis, it was observed that there was no statistically significant difference in the rate of re-intervention or re-do surgery between non-obese and obese patients who underwent LARS (OR: 0.52, 95% CI: 0.23 to 1.16, p = 0.11) [12-14,18-19] (Figure 6). This analysis was based on studies that investigated the need for re-intervention or re-do surgery following LARS in both patient groups. Interestingly, the rate of endoscopic dilatation, a standard measure for managing post-operative dysphagia, was found to be similar among both obese and non-obese patients.

**Figure 7:**
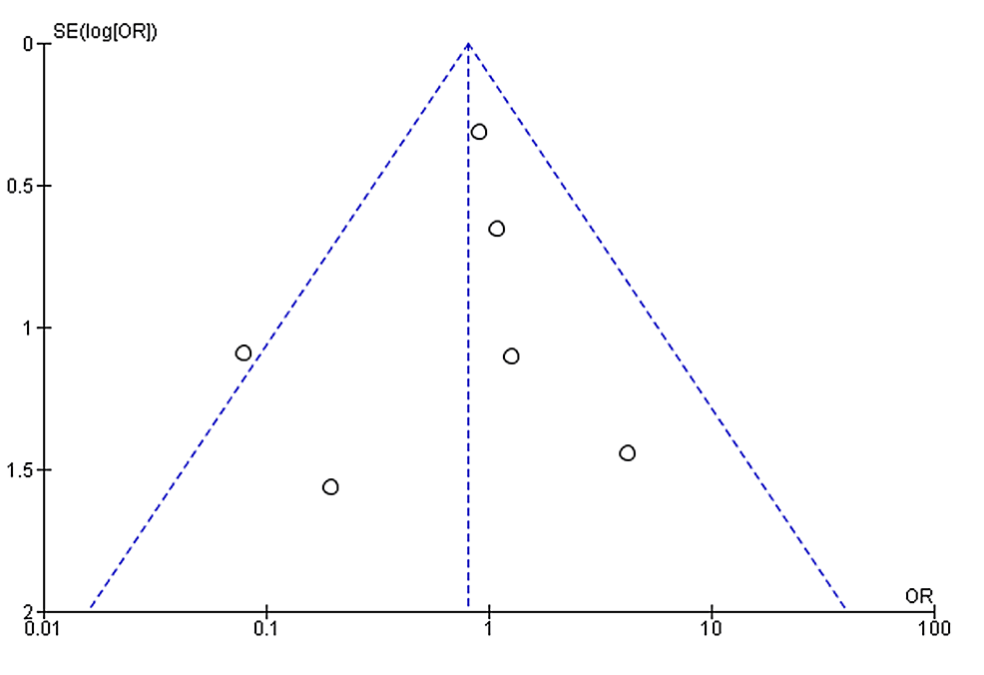
Funnel plots for the studied outcomes were symmetrical, suggesting the absence of publication bias

It is worth noting that literature reports have raised concerns about a potentially higher rate of post-operative dysphagia after LNF compared to partial fundoplication procedures, which could result in a need for post-operative re-intervention. However, a subgroup analysis to assess this specific concern could not be performed due to the diverse range of operative approaches reported in the included studies. This aspect introduces the potential for confounding factors and bias in our study's reported rate of endoscopic dilatations. Furthermore, the funnel plot displaying a symmetrical distribution suggests the absence of publication bias (Figure 7).

Early Return to Theatre

**Figure 8:**
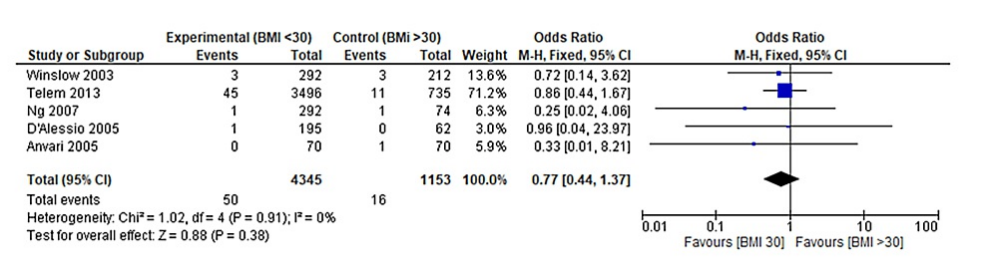
Forest plot on early return to operating theatre post-LARS in non-obese and obese patients Ng et al. [13], Winslow et al. [14], D'Alessio et al. [15], Telem et al. [17], Anvari & Bamehriz [18] LARS: Laparoscopic anti-reflux surgery

Upon conducting a meta-analysis of the five studies that investigated the occurrence of early return to the operating theatre after LARS, the findings demonstrated no statistically significant difference between non-obese and obese patients (OR: 0.77, 95% CI: 0.44 to 1.37, p = 0.38) (Figure 8). This analysis, encompassing [13-15,17-18] studies, underscores that the likelihood of early surgical re-intervention did not significantly differ based on the patient's obesity status.

**Figure 9:**
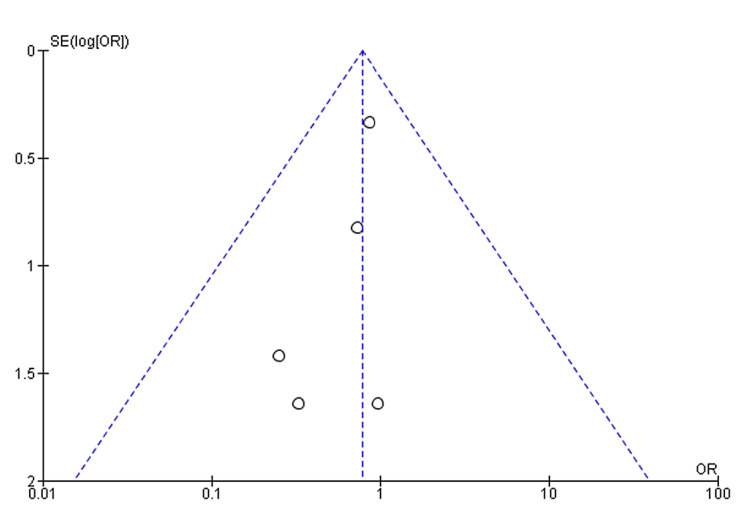
Funnel plots for the studied outcomes were symmetrical, suggesting the absence of publication bias

The absence of a significant difference in this outcome suggests a balanced risk profile for both non-obese and obese patients in terms of requiring an early return to the operating theatre post-LARS. These findings contribute valuable insights to the comprehensive understanding of the surgical outcomes in both patient groups. Furthermore, the funnel plot displaying a symmetrical distribution suggests the absence of publication bias (Figure 9).

Heterogeneity and Publication Bias Assessment

In the assessment of statistical heterogeneity across the conducted analyses, it was found that no significant heterogeneity was observed (refer to Table 2 for details). Notably, this lack of substantial variation within the collected data enhances the reliability and consistency of the study outcomes.

Moreover, the analysis of funnel plots for the studied outcomes revealed a symmetrical distribution. This symmetry in the funnel plots suggests a lack of publication bias, further reinforcing the robustness and impartiality of the findings. The absence of significant heterogeneity and publication bias adds to the credibility of the study's results and their applicability in informing clinical practice and decision-making.

Discussion

The adoption of LARS as the definitive treatment for GERD is now universally acknowledged [2,3]. The prevalence of obesity has surged significantly, with up to 30% of individuals in the Western world classified as obese [1], posing a pivotal challenge for medical interventions. The initial investigation into the impact of obesity on LARS outcomes by Perez et al., opting for a thoracic approach to mitigate the effects, yielded contrary results: obesity negatively influenced both trans-thoracic and trans-abdominal procedures [21].

Subsequent studies on the effects of obesity on LARS produced a diverse range of outcomes, leading to conflicting interpretations [12,16,18-20]. Our systematic review and meta-analysis aim to untangle these inconsistencies. Classifying patients based on WHO criteria into obese and non-obese groups, we initially hypothesized more significant technical challenges and increased peri- and post-operative complications in obese patients [21]. Astonishingly, our study revealed contrasting results, indicating comparable adverse outcomes between the two BMI groups. This could be attributed to advancements in risk assessment, anesthesia techniques, pain management, and surgical procedures over the past two decades.

The most pivotal discovery from our meta-analysis is the heightened risk of reflux recurrence following LARS in obese patients. Our findings unveiled a significantly higher incidence of reflux symptoms recurrence, with a pooled rate of 9.5% in obese patients, in contrast to 3.04% in non-obese patients, within an average follow-up of 35 months. This outcome questions the long-term efficacy of repair within the obese cohort. While individual studies [12,16,18] may not have individually demonstrated this elevated risk, our collective analysis elucidated a statistically significant increase in recurrence among obese patients. A possible explanation for this is the augmented intra-abdominal pressure observed in obesity, which may displace the lower oesophageal sphincter and disrupt the wrap integrity, possibly leading to migration into the chest. Furthermore, vagal abnormalities inherent to obesity might exacerbate the situation by increasing bile and pancreatic enzyme secretion [20]. 

This discussion leads to the pertinent question: How can we mitigate the risk of reflux recurrence in obese patients? Existing literature suggests that weight loss significantly improves reflux symptoms [22]. Hence, preoperative weight loss strategies could be considered an option to alleviate symptoms and curb recurrence. Alternatively, Roux-en-Y (R&Y) gastric bypass, a bariatric procedure with added benefits in managing GERD symptoms, is a viable avenue [22-23].

Critics might advocate for randomized controlled trials (RCTs) to provide level 1a evidence for addressing this issue. However, the paucity of RCTs in this domain necessitates including non-randomized studies in our meta-analysis. As indicated by the Meta-analysis of Observational Studies in Epidemiology (MOOSE) guidelines, the consensus among researchers supports incorporating observational studies in meta-analyses [24]. This recognition underscores the importance of our study in advancing our understanding of LARS outcomes about obesity despite the limitations imposed by the available data [24].

Study Limitations

Several inherent limitations impact the comprehensiveness and robustness of our study. Foremost among these is the methodological quality of the included research papers. Primarily stemming from single centers, most of these studies adopt a retrospective design, inherently introducing biases contributing to the observed heterogeneity within the pooled data. Notably, five of the incorporated scientific papers are retrospective cohort studies, which inherently carry a risk of confounding bias. Furthermore, the choice of operative technique varied among surgeons, and the definition of reflux recurrence needed to be more consistently required to be more consistently applied across studies, resulting in diverse post-operative outcomes.

Crucially, the incidence of post-operative symptoms, such as food intolerance, dysphagia, and belching, as well as the ongoing use of anti-reflux medications, lacked comprehensive and accurate reporting in the selected papers. Additionally, caution is warranted when interpreting results for severely obese patients with a BMI >40 kg/m², as all patients with a BMI >30 kg/m² were grouped in a single obese category, potentially obscuring nuanced differences.

Furthermore, our analysis of total operative time revealed significant disparity across reported studies, attributed to varying definitions of this parameter. This ambiguity underscores the need for standardized reporting in future research.

## Conclusions

Our study's findings underscore a high BMI (>30) as a significant risk factor for reflux recurrence following anti-reflux surgery (ARS). We recommend that, while laparoscopic anti-reflux surgery (LARS) can be performed safely in obese patients, the evidence substantiates comparable peri- and post-operative outcomes to non-obese patients. However, a notable caveat emerges in the form of a substantial recurrence rate of up to 9.5% within the initial 35 months post-surgery. This highlights the urgency of further exploration through well-designed, prospective multicentre trials, an avenue currently underrepresented in the literature.

Notably, bariatric procedures such as Roux-en-Y (R&Y) gastric bypass hold a recognized role in gastroesophageal reflux disease (GERD) management among the morbidly obese, exhibiting symptom improvement. In the interim, clinicians are advised to exercise caution when offering LARS to obese patients, with weight reduction strategies being a prudent recommendation. The severity of the disease must be weighed against the surgical risks in obese patients, with the benefits ultimately outweighing the potential hazards.

## References

[1] El-Serag HB, Sweet S, Winchester CC, Dent J (2014). Update on the epidemiology of gastro-oesophageal reflux disease: a systematic review. Gut.

[2] Lundell L (2014). Borderline indications and selection of gastroesophageal reflux disease patients: 'Is surgery better than medical therapy'?. Dig Dis.

[3] Lundell L, Miettinen P, Myrvold HE (2001). Continued (5-year) followup of a randomized clinical study comparing antireflux surgery and omeprazole in gastroesophageal reflux disease. J Am Coll Surg.

[4] Broeders JA, Roks DJ, Ahmed Ali U (2013). Laparoscopic anterior 180-degree versus nissen fundoplication for gastroesophageal reflux disease: systematic review and meta-analysis of randomized clinical trials. Ann Surg.

[5] Friedenberg FK, Xanthopoulos M, Foster GD, Richter JE (2008). The association between gastroesophageal reflux disease and obesity. Am J Gastroenterol.

[6] Barak N, Ehrenpreis ED, Harrison JR, Sitrin MD (2002). Gastro-oesophageal reflux disease in obesity: pathophysiological and therapeutic considerations. Obes Rev.

[7] Abdelrahman T, Latif A, Chan DS (2018). Outcomes after laparoscopic anti-reflux surgery related to obesity: a systematic review and meta-analysis. Int J Surg.

[8] Bashir Y, Chonchubhair HN, Duggan SN (2019). Systematic review and meta-analysis on the effect of obesity on recurrence after laparoscopic anti-reflux surgery. Surgeon.

[9] Moher D, Liberati A, Tetzlaff J, Altman DG (2009). Preferred reporting items for systematic reviews and meta-analyses: the PRISMA statement. PLoS Med.

[10] Body Mass Index . https://www.cdc.gov/healthyweight/assessing/bmi (2023). Body mass index. https://www.cdc.gov/healthyweight/assessing/bmi/.

[11] Julian PT Higgins, Sally Green (2011). Cochrane handbook for systematic reviews of interventions. Cochrane handbook for Systematic Reviews of Interventions, John Wiley & Sons.

[12] Tekin K, Toydemir T, Yerdel MA (2012). Is laparoscopic antireflux surgery safe and effective in obese patients?. Surg Endosc.

[13] Ng VV, Booth MI, Stratford JJ, Jones L, Sohanpal J, Dehn TC (2007). Laparoscopic anti-reflux surgery is effective in obese patients with gastro-oesophageal reflux disease. Ann R Coll Surg Engl.

[14] Winslow ER, Frisella MM, Soper NJ, Klingensmith ME (2003). Obesity does not adversely affect the outcome of laparoscopic antireflux surgery (LARS). Surg Endosc.

[15] D'Alessio MJ, Arnaoutakis D, Giarelli N, Villadolid DV, Rosemurgy AS (2005). Obesity is not a contraindication to laparoscopic Nissen fundoplication. J Gastrointest Surg.

[16] Tsuboi K, Omura N, Yano H, Kashiwagi H, Kawasaki N, Suzuki Y, Yanaga K (2009). Body mass index has no effect on the results of laparoscopic fundoplication in Japanese patients with reflux esophagitis. Esophagus.

[17] Telem DA, Altieri M, Gracia G, Pryor AD (2014). Perioperative outcome of esophageal fundoplication for gastroesophageal reflux disease in obese and morbidly obese patients. Am J Surg.

[18] Anvari M, Bamehriz F (2006). Outcome of laparoscopic Nissen fundoplication in patients with body mass index ≥35. Surg Endosc.

[19] Schietroma M, Piccione F, Clementi M (2017). Short- and long-term, 11-22 years, results after laparoscopic Nissen fundoplication in obese versus nonobese patients. J Obes.

[20] Andolfi C, Vigneswaran Y, Kavitt RT, Herbella FA, Patti MG (2017). Laparoscopic antireflux surgery: importance of patient's selection and preoperative workup. J Laparoendosc Adv Surg Tech A.

[21] Perez AR, Moncure AC, Rattner DW (2001). Obesity adversely affects the outcome of antireflux operations. Surg Endosc.

[22] Frezza EE, Ikramuddin S, Gourash W, Rakitt T, Kingston A, Luketich J, Schauer P (2002). Symptomatic improvement in gastroesophageal reflux disease (GERD) following laparoscopic Roux-en-Y gastric bypass. Surg Endosc.

[23] Nelson LG, Gonzalez R, Haines K, Gallagher SF, Murr MM (2005). Amelioration of gastroesophageal reflux symptoms following Roux-en-Y gastric bypass for clinically significant obesity. Am Surg.

[24] Stroup DF, Berlin JA, Morton SC (2000). Meta-analysis of observational studies in epidemiology: a proposal for reporting. Meta-analysis Of Observational Studies in Epidemiology (MOOSE) group. JAMA.

